# A Novel Variant in the FBN1 Gene Causing Marfan Syndrome: A Case Report

**DOI:** 10.7759/cureus.56948

**Published:** 2024-03-26

**Authors:** Gabriel A Jiménez-Berríos, Sebastián J Vázquez-Folch, Natalio Izquierdo

**Affiliations:** 1 Department of Ophthalmology, School of Medicine, Universidad Central del Caribe, Bayamón, PRI; 2 Department of Surgery, School of Medicine, Medical Sciences Campus, University of Puerto Rico, San Juan, PRI

**Keywords:** camptodactyly, fibrillin, autosomal dominant, ectopia lentis, marfan syndrome

## Abstract

Our purpose is to report a patient with a novel variant in the fibrillin-1 (*FBN1*)* *gene causing the Marfan syndrome (MFS).

The 29-year-old female patient with musculoskeletal, cardiovascular, and ocular findings compatible with the MFS had a novel pathogenic mutation on the *FBN1* gene.

We report on a patient whose clinical findings are compatible with the MFS. This patient’s variant on the *FBN1* gene leading to the syndrome has not been previously described. Additional investigations are needed to determine whether this variant contributes to the development of camptodactyly in patients with the syndrome.

## Introduction

Marfan syndrome (MFS) is a common hereditary condition that particularly affects the body's connective tissue. It has a prevalence of around 1 in 5,000 people [1]. Patients with MFS have a constellation of clinical features and a heterogeneous phenotype. Nowadays, patients are diagnosed using the revised Ghent criteria [1] and genetic findings. Several physical characteristics have been identified and used to establish a clinical diagnosis. Clinical systemic manifestations include [2] musculoskeletal, cardiovascular, and ophthalmic.

Musculoskeletal signs include tall stature, long extremities, arachnodactyly dolichostenomelia, positive wrist and ulnar signs, camptodactyly [3], joint hypermobility, pectus excavatum and/or carinatum, maxillary hypoplasia, and gothic palate. Cardiovascular manifestations in patients with the syndrome include aortic root dilation and dissection, mitral valve prolapse, and regurgitation [4]. Ocular manifestations in patients with the syndrome include ectopia lentis [5], elongated axial length, strabismus [6], cornea plana, glaucoma [7], and retinal detachment [8].

The MFS has an autosomal dominant inheritance. Dietz and co-workers first described the fibrillin-1 (*FBN1*) gene associated with the syndrome [9]. There are over 2000 published *FBN1* variants and many are unique to individual families. Missense variants, especially cysteine (Cys) substitutions, are the most common type of *FBN1* variant [10]. Identifying the specific variation within the *FBN1 *gene and assessing its probability of being pathogenic are crucial elements in diagnosing MFS. Variants such as de novo mutations (occurring without a family history) as well as those involving nonsense, frameshift, splicing, and missense substitutions at conserved sites are typically regarded as having higher likelihoods of being pathogenic. [11].

We report on a patient with a novel variant in the *FBN1* gene who has systemic manifestations such as camptodactyly and ectopia lentis as part of the MFS.

## Case presentation

**Figure 1:**
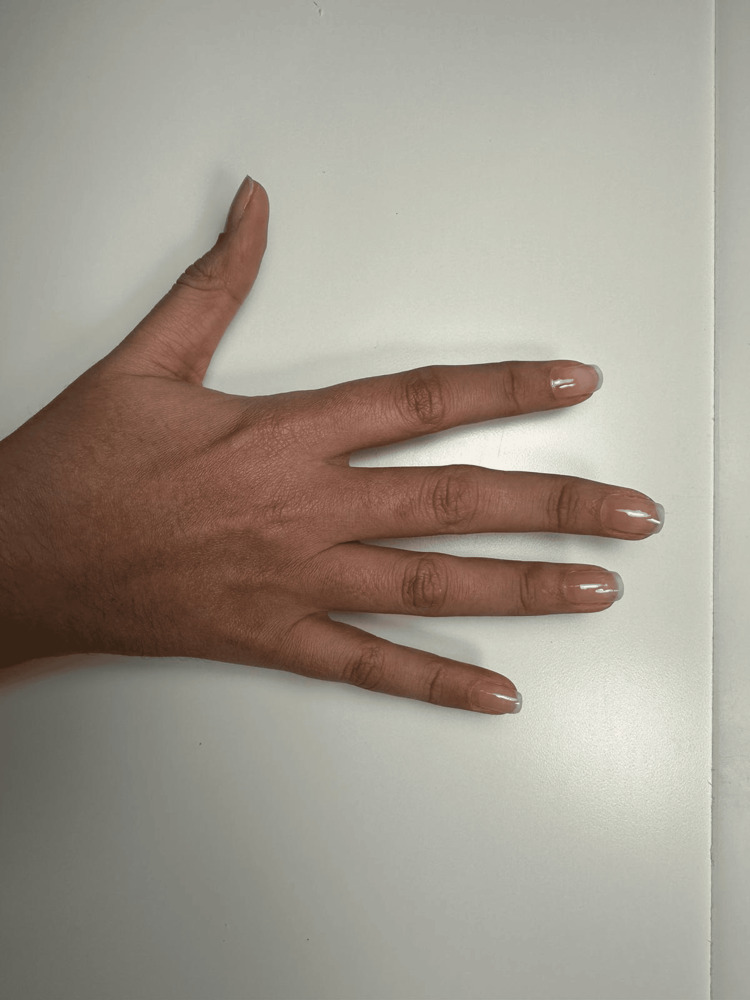
Camptodactyly right hand

**Figure 2:**
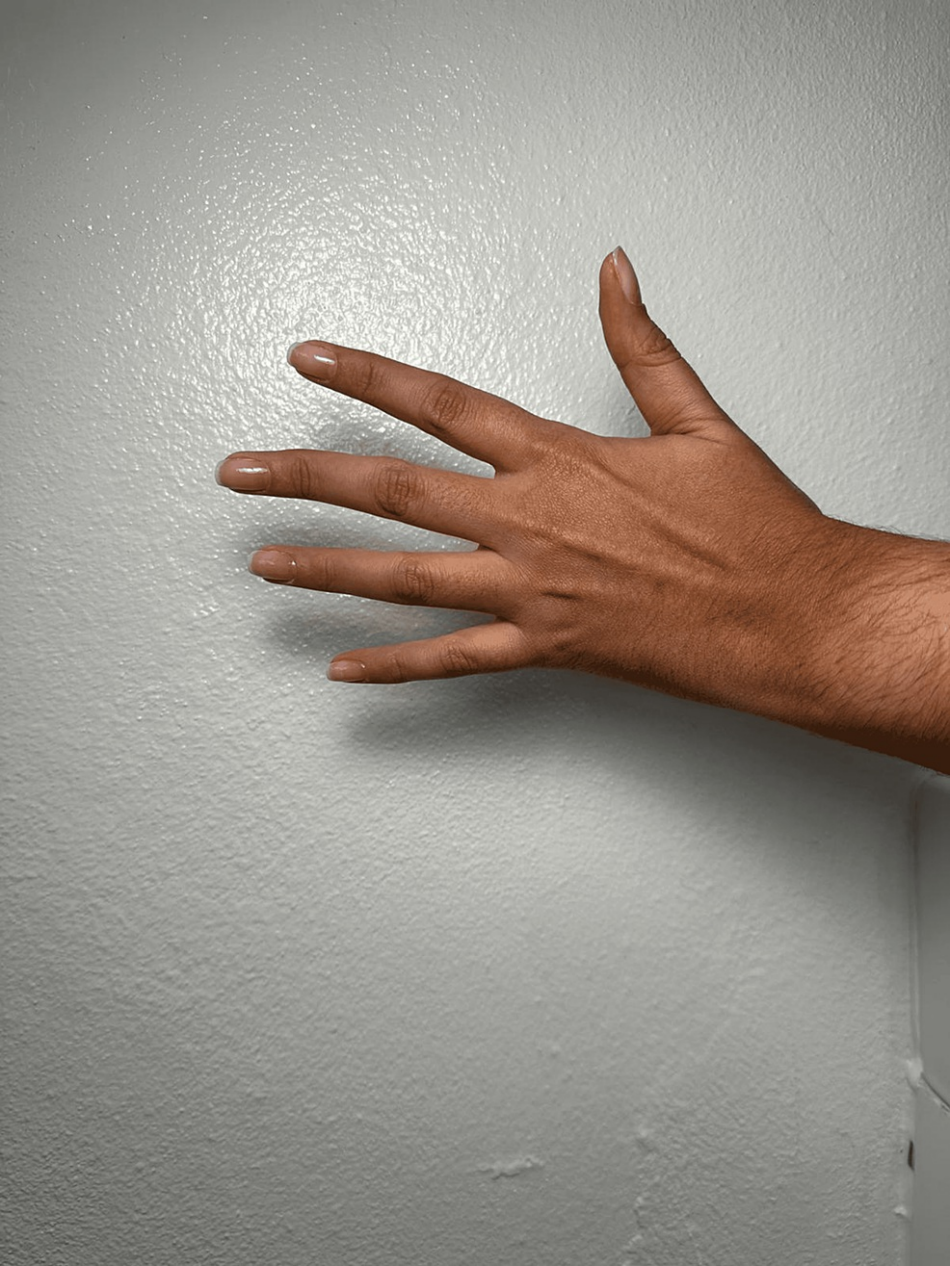
Camptodactyly left hand

A 28-year-old female patient was referred for ophthalmic evaluation due to a mitral valve prolapse. Upon physical examination, the patient had pectus excavatum, long extremities, dolichostenomelia, a positive wrist sign, a positive ulnar sign, arachnodactyly, camptodactyly of both hands, as depicted in Figures 1, 2, malar hypoplasia, blue sclera, and a gothic palate.

The patient underwent a comprehensive ophthalmic evaluation by at least one of the authors (NJ Izquierdo). The best corrected visual acuity was 20/20-1 and 20/25, in the right and left eye, respectively. Refraction was -2.50DS +1.00DC x 105˚ and -3.00DS +1.25DC x 75˚ in the right and left eye, respectively. Upon slit lamp examination, the patient had a smooth velvety iris without iridodonesis. The patient had minimal upward lens dislocation in the left eye only.

Upon indirect ophthalmoscopy, the patient had asymmetric cupped optic nerves, intact vessels, maculae, and peripheries.

**Figure 3:**
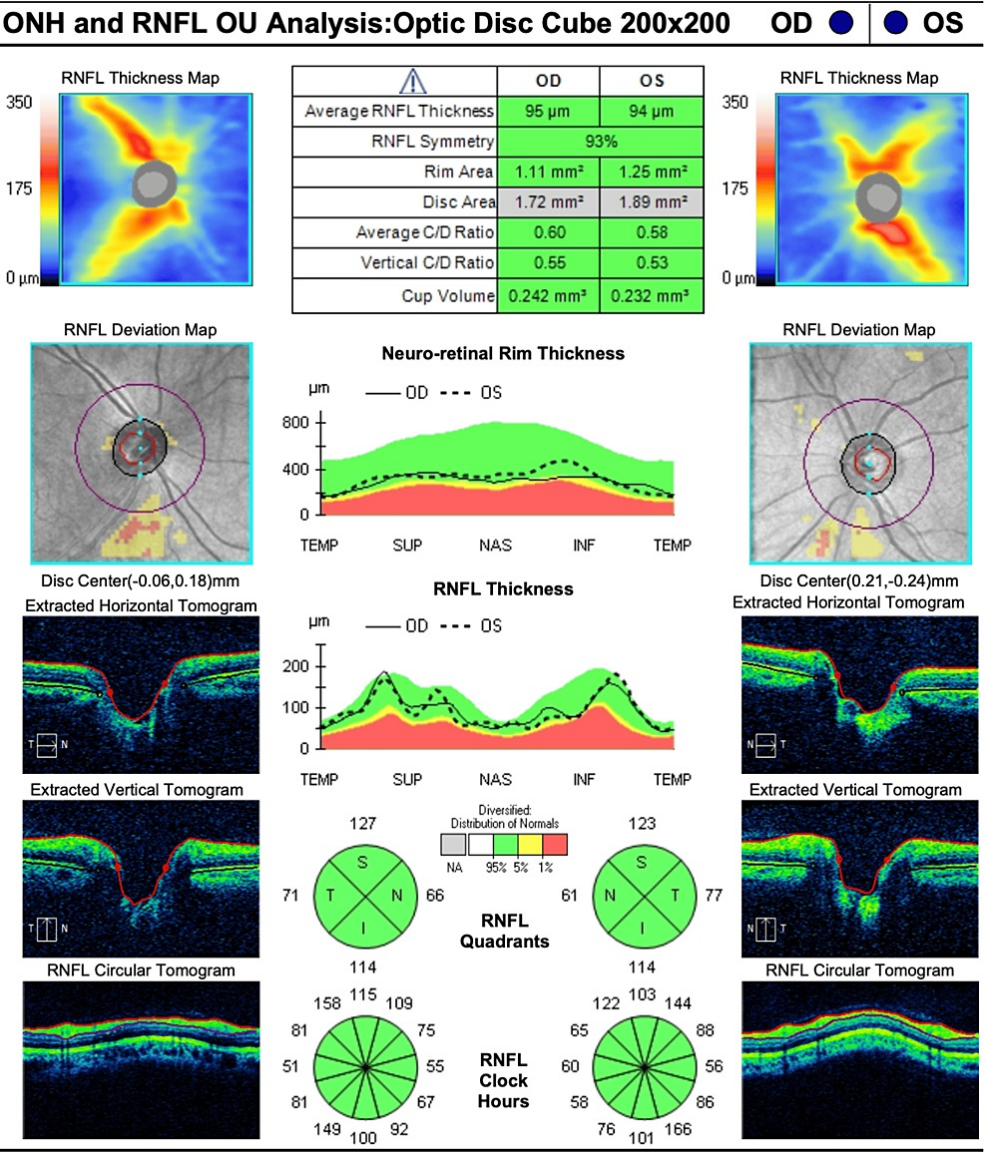
Patient's optic coherence tomography RNFL: Retinal nerve fiber layer; ONH: Optic nerve hypoplasia; OD: Right eye; OS: Left eye

Upon optic nerve coherence tomography (Carl Zeiss Meditec, Inc., Dublin, United States), the patient had a retinal fiber layer of 95 mm and 94 mm; and the average cup-to-disk ratio was 0.60 and 0.58 in the right and left eye, respectively, as presented in Figure 3.

Visual field testing (30-2 Carl Zeiss Meditec, Inc.) showed a mean deviation of -1.92 dB (p<10%) and -1.80 dB (p<10%) in the right and left eye, respectively.

The patient is currently not using any systemic medications. Being a glaucoma suspect she is not being prescribed any ophthalmic medications.

*FBN1* full gene sequencing was done (Laboratory for Molecular Medicine, Center for Genetics and Genomics, Cambridge, United States). It showed a heterozygous mutation in the *FBN1* with a novel presumed pathogenic variant c.4001G>A (p.Gly1334Asp) on exon 32.

## Discussion

Previous studies have reported that patients with MFS have several musculoskeletal findings [9]. Our patient had pectus excavatum, long extremities, dolichostenomelia, positive wrist sign, positive ulnar sign, arachnodactyly, and camptodactyly of both middle fingers. These findings are compatible with previous literature [1,3,9].

Zeigler and co-workers have reported that patients with MFS have cardiovascular manifestations, including aortic root dilation and dissection, mitral valve prolapse, and aortic insufficiency [12]. Our patient had mitral-valve prolapse. Her cardiovascular findings are compatible with the MFS.

Ocular findings in patients with MFS have been described extensively [5]. Our patient had myopia, blue sclerae, smooth velvety iris, ectopia lentis, and optic nerve cups. These findings are compatible with previous studies.

Glaucoma has been reported in patients with the syndrome [5,7]. The suspicion of glaucoma was prompted by the asymmetry observed in our patient's optic nerve cups. However, visual field test mean deviation results were within normal limits. Glaucoma evaluation in all patients with MFS is needed. 

Several mutations in the *FBN1* gene have been associated with MFS [13]. Our patient had heterozygosity for a specific *FBN1* mutation (c. 4001G>A (p.Gly1334Asp)). To our knowledge, scant data has been reported on this variant in the *FBN1* gene leading to the syndrome. This variant has not been reported in Puerto Rico.

Yoon and Kong [3] first reported camptodactyly in patients with MFS. As depicted in Figures 1, 2, our patient had camptodactyly of the middle finger of both hands. One could hypothesize that this variant of the *FBN1* gene may be linked to the development of camptodactyly in patients with the syndrome. Nonetheless, further comprehensive research is necessary to thoroughly investigate this potential association.

This case also raises the intriguing possibility that the inheritance mechanisms governing specific ophthalmic findings may be more complex than previously thought.

## Conclusions

We report on a patient whose clinical findings are compatible with MFS. This patient’s variant on the *FBN1 *gene leading to the syndrome has scant data. Additional investigations are needed to determine whether this variant contributes to the development of camptodactyly in patients with the syndrome.
